# A Case of Squamous Cell Carcinoma of Conjunctiva as Initial Sign of Systemic Cancers

**DOI:** 10.1155/2011/972318

**Published:** 2011-12-29

**Authors:** Hayato Mitamura, Toshiyuki Oshitari, Ryuta Kimoto, Jiro Yotsukura, Kaoru Asanagi, Takayuki Baba, Yoko Takahashi, Takashi Kishimoto, Shuichi Yamamoto

**Affiliations:** ^1^Department of Ophthalmology and Visual Science, Chiba University Graduate School of Medicine, Chuo-ku, Chiba 260-8670, Japan; ^2^Department of Pathology, Chiba University Graduate School of Medicine, Chuo-ku, Chiba 260-8670, Japan; ^3^Department of Molecular Pathology, Chiba University Graduate School of Medicine, Chuo-ku, Chiba 260-8670, Japan

## Abstract

The purpose of this report is to present the findings in a case of squamous cell carcinoma (SCC) of the conjunctiva which was the initial sign of systemic cancers. A 94-year-old woman without known systemic diseases developed a mass in her right conjunctiva. She was referred to our hospital 5 months after the onset. She was diagnosed with conjunctival SCC by biopsy. Systemic CT before the surgery revealed multiple liver lesions, lung legions, and a large mass surrounding the appendix. The patient requested the surgery, and the main aim of the surgery was cosmesis. Histopathological examinations of the specimen led us to the final diagnosis as SCC. She did not receive any other therapy because of her age. As no other surgical procedures were undertaken, it is uncertain as to whether the conjunctival lesion was primary or secondary. Although, it is extremely rare that SCC of the conjunctiva is the initial sign of systemic cancers, careful systemic examinations to find other cancers should be made.

## 1. Introduction

A squamous cell carcinoma (SCC) of the conjunctiva accompanied with systemic cancers is very rare [1]. Only one case has been reported where a conjunctival SCC was the initial finding in a patient with systemic cancer [2]. We report a rare case of SCC of the conjunctiva as the initial manifestation of systemic cancers.

## 2. Case Report

A 94-year-old woman had noticed that the conjunctiva of the right eye was hyperemic 10 months earlier. She was suspected of having a conjunctival tumor at a private clinic 5 months later but she refused to undergo further examinations because of her age. After a rapid increase in the size of the mass, she was referred to the Chiba University Hospital.

**Figure 1:**
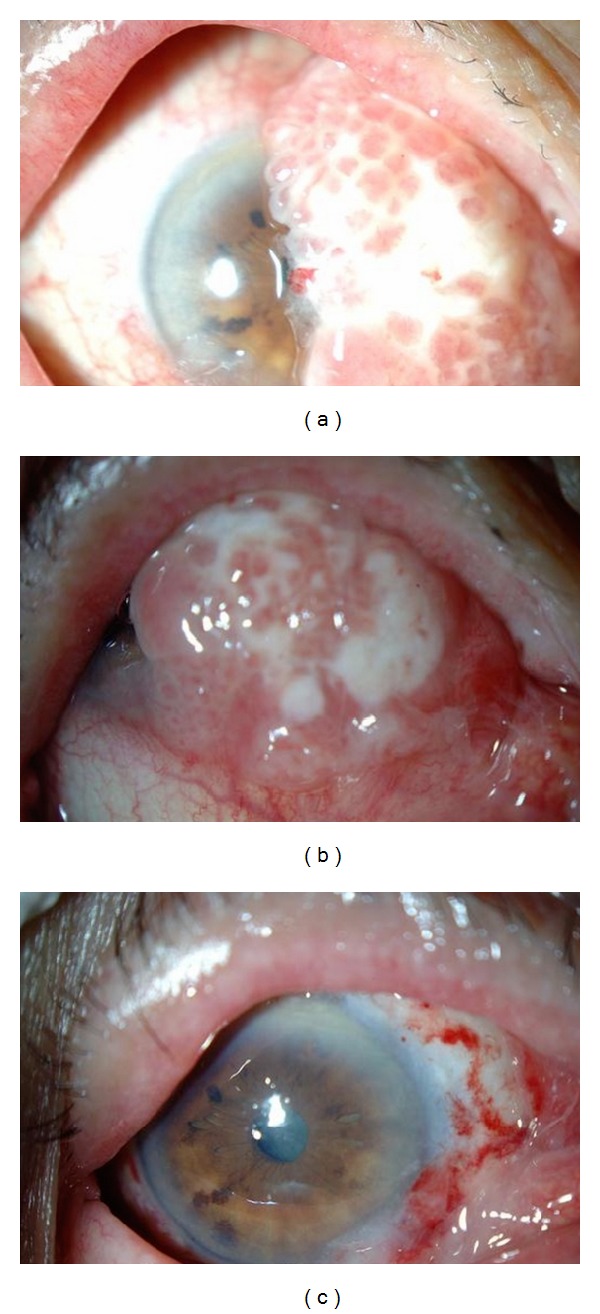
Photograph of the eye showing a large mass at the limbus that extended over the pupil (a and b) and postoperative photograph of the slit-lamp examination (c). Conjunctival tumor was almost excised with the nasal part of conjunctiva. No recurrence was observed until her death.

 At the first examination, her visual acuities were hand motion at 30 cm OD and 0.8 OS. Slit-lamp examination showed a large, irregular-surface mass on the nasal conjunctiva. The mass was so large that it covered the pupil of the eye (Figures 1(a) and 1(b)). The ocular movements were full in both eyes. The eye was not proptosed and the eyelids were not retracted. She had neither history nor symptoms or signs of systemic cancers. Cytopathological examination from a scraping biopsy showed a class IV, well-differentiated SCC. 

**Figure 2:**
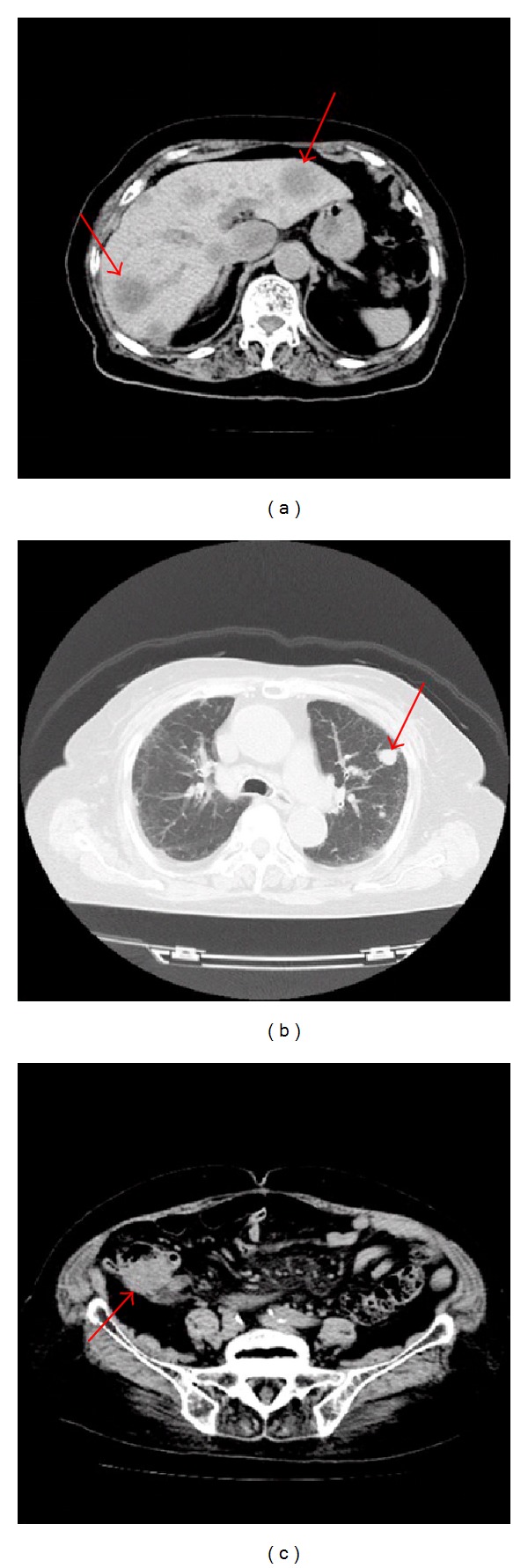
Images of systemic CT examination from the liver (a), the lung (b), and the abdomen (c). The arrow surrounding the appendix in the panel (c) may indicate the primary site of the cancer. The arrows in (a) show multiple metastasis of the liver. The arrow in (b) indicates the metastasis of the lung.

 Because the size of the tumor was large, we performed a systemic CT examination. The CT examination showed a large tumor surrounding the appendix, multiple legions in the liver, and lungs (Figure 2). No other orbital lesions were found in the CT examination. 

**Figure 3:**
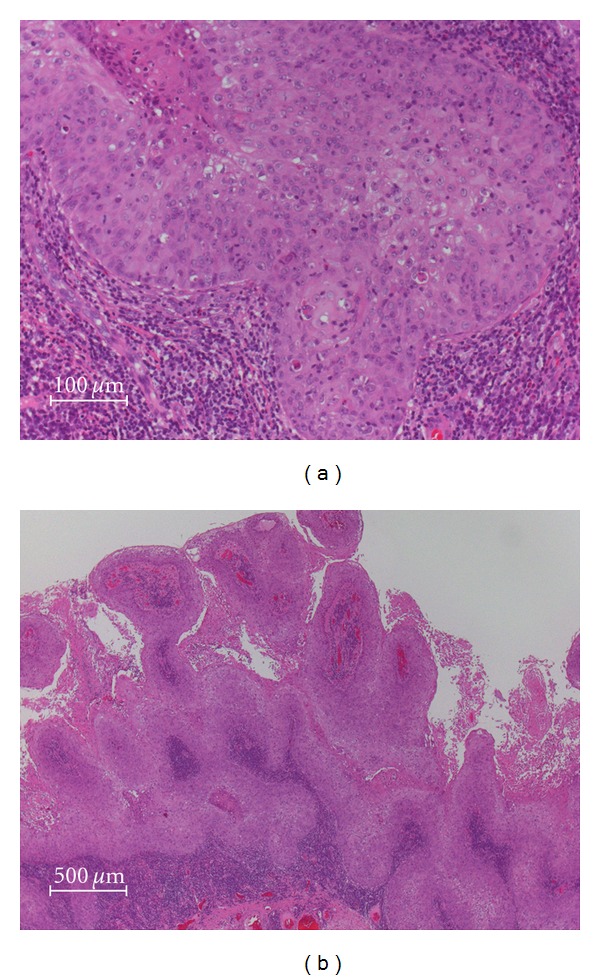
Histopathology of conjunctival tumor. The mass was composed of the sheet-like proliferation of atypical stratified squamous cells with outstanding papillary constructed growth pattern. The pleomorphic squamous cells showed numerous mitotic figures with dyskaratosis, loss of the nuclear polar, and lucid nucleolus. The histopathological diagnosis was well-differentiated type of squamous cell carcinoma.

 Because the patient requested surgery for cosmesis, we excised the tumor and used 0.04% Mitomycin C eyedrops 2 times/day. The tumor was almost completely excised (Figure 1(c)), and the patient and her family were very happy with the results of the operation. Histopathological examination of the specimen showed a well-differentiated SCC of the conjunctiva (Figure 3). She declined treatment for the primary systemic cancer because of her age. The patient died of the primary systemic cancer one month after our surgery.

## 3. Discussion

Cervantes et al. reviewed 287 cases of SCC of the conjunctiva, and only two cases had regional metastasis (0.7%) [3]. Grossniklaus et al. reviewed 2,455 cases of conjuctival lesions and only one case had a metastasized mass (0.04%) [1]. Thus, it is rare that a conjunctival SCC is accompanied with systemic cancers. In our case, we performed systemic CT examinations because the size of the tumor was relatively large. As a result, we found masses in the lungs, liver, and surrounding the appendix. We did not determine whether a SCC of conjunctiva in our case is a conjunctival metastasis because we had no chance to perform biopsy for systemic cancers. Although it is a speculation, it is likely from the size of the lesions that the tumor surrounding the appendix may be the primary site. The most common type of appendix tumor is carcinoid tumor followed by adenocarcinoma, but recent reports indicate that carcinoid tumor can be combined with SCC [4, 5]. However, we could not rule out the possibility that the conjunctival SCC was a primary tumor and the lung and the liver metastases were derived from the tumor surrounding the appendix.

 The patient did not undergo chemotherapy because of her age, and the purpose of the operation was cosmetic. However, the patient and her family were very satisfied with the outcome of our surgery. Thus, even when the life span cannot be extended by a surgery, the quality of life can be significantly improved. Thus, although the patient died one month after the operation, we believe that our decision of performing tumor excision in this case was appropriate and meaningful for the patient and her family.

 It is extremely rare that a conjunctival tumor is the initial manifestation of systemic cancers [2]. More commonly, patients with a conjunctival tumor have a history of a primary cancer [6, 7]. Our findings indicate that even if a conjunctival SCC as the initial finding of systemic cancer is extremely rare, systemic examinations should be considered to rule out the existence of systemic cancers.
